# Stress produces negative judgement bias in cuttlefish

**DOI:** 10.1098/rsbl.2024.0228

**Published:** 2024-10-09

**Authors:** Sarah E. Giancola-Detmering, Robyn J. Crook

**Affiliations:** ^1^ Department of Biology, San Francisco State University, 1600 Holloway Avenue, San Francisco, CA, USA

**Keywords:** affective state, cephalopod, handling, husbandry, cognitive bias, welfare

## Abstract

Judgement bias tasks (JBTs) are used to assess the emotional state and welfare of animals in zoos, farms and laboratories, based on the interpretation of an ambiguous or intermediate cue. Animals in positive affective states are more likely to interpret the ambiguous cue positively, whereas animals experiencing negative affect are more likely to interpret ambiguous cues pessimistically. Here, we developed a modified JBT assay for the stumpy-spined cuttlefish, *Sepia bandensis*, to determine whether cuttlefish exhibit negative affective states resulting from external stressors. Positive and neutral visual cues were presented twice daily until animals learned to associate food with the reinforced visual cue. After training, one treatment group was exposed to combined exposure and handling stress produced by 6 days of impoverished housing and simulated net capture. Our control group received no stress experience. In test trials performed after the stress experience, stressed animals showed higher latencies to approach ambiguous cues, spent significantly less time in rooms with ambiguous cues once they entered, and were less likely to enter first into the ambiguous cue-paired room compared with controls. These behaviours suggest that stress induces pessimistic judgement bias in cuttlefish, the first indication of this capacity in cephalopods.

## 1. Introduction

The use of cephalopods as model animals in biological research is growing rapidly. Their complex nervous systems and unique behaviours have made them ideal research models for neurobiology, behaviour and ecology. With increased popularity, there is a pressing need for improved ways to assess stress and welfare [1].

One way of examining welfare is to use cognitive bias tests (also known as judgement bias tasks (JBTs)) to assess affective state based on responses to an ambiguous cue [2]. Typically, animals are trained to associate a positive cue with a rewarding outcome (i.e. food) and neutral/negative cue with no outcome (no food). During the test phase, an ambiguous cue, intermediate between the positive and neutral/negative cue, is presented. Theory and empirical findings indicate that individuals in positive affective states are more likely to behave as if expecting a reward (an ‘optimistic’ behavioural response) than those in more negative states who show a ‘pessimistic’ behavioural response [3,4].

JBTs have been used to assess affective state and welfare in many vertebrates such as rats, mice, dogs, zebrafish and pigs [5–9]. More recently, evidence is emerging that cognitive or judgement bias can be found in some invertebrates such as insects [10,11]. However, the use of JBTs in invertebrates remains uncommon, and to date, JBTs have not been performed on any cephalopod.

Although previous studies of affective state in cephalopods have suggested the presence of both negative affect resulting from pain [12] and positive affect resulting from unexpected reward [13], there have been no investigations of the effect of stress on affect or welfare. Stressful experiences cause long-lasting changes to physiology in cephalopods [14], but little is known about how housing, handling and management of cephalopods in captivity may produce changes to affect. In cuttlefish, impoverished housing slows the growth rate and negatively affects memory and cognition [15]. Similar work suggests that an enriched environment improves cryptic coloration [16] and increases hunting success [17], suggesting that housing is critical to good health, but direct assessments of welfare remain scarce.

Chronic and acute stressors negatively affect vertebrate animals in captivity, causing cognitive and behavioural changes [3]. Various behaviours reveal stress in vertebrate animals: repeated movements [18], decreased exploratory behaviour [19], excessive grooming [20] and changes in social and sexual behaviour [21]. Multiple studies have shown that vertebrate animals exposed to stressful stimuli, enrichment removal and unpredictable housing conditions respond negatively (or pessimistically) to an ambiguous cue during JBTs [2,22], indicating both that these events are stressful and that JBTs are an appropriate assay to capture animals’ experiences of them [23].

In this study, we used a modified JBT with simultaneous discrimination training to assess judgement bias associated with combined housing and handling stress. Cuttlefish were trained to approach paddles marked with either vertical or horizontal stripes for a food reward and not approach a neutral paddle (marked with stripes in the opposite orientation to the positive paddle), which signalled no reward [2]. Given the large volume of studies using simultaneous discrimination of visual cues to direct cuttlefish behaviour [24–27], we expected that this task would be learned successfully by our animals. We used a combination of prolonged exposure stress and transient, repeated handling stress to attempt to produce a negative affective state and assessed whether these treatments produced evidence for pessimistic judgement bias.

## 2. Material and methods

### (a) Animals

**Figure 1.**
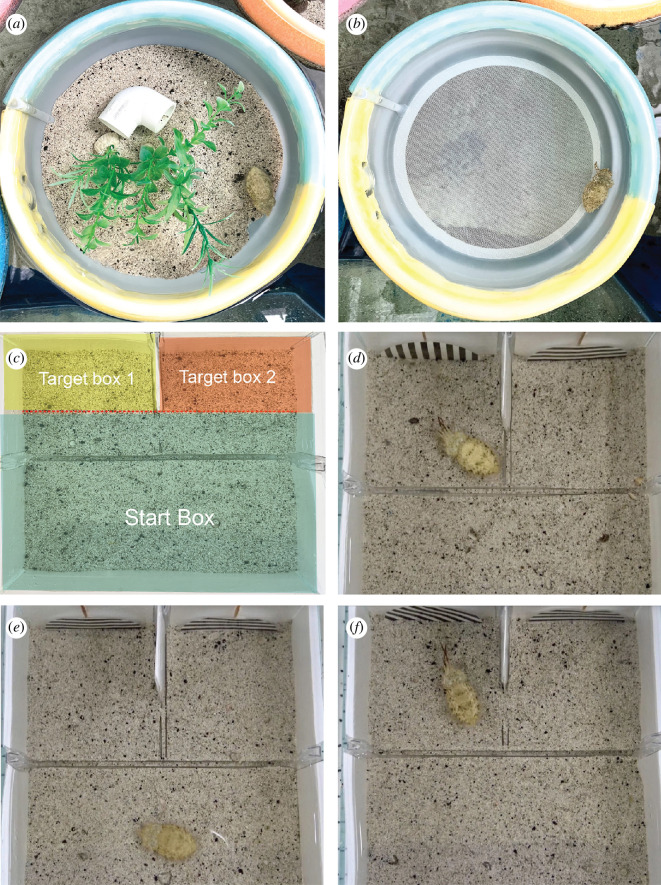
(*a*) A standard, enriched housing set-up used as our control condition. (*b*) Housing for treatment animals with impoverished housing. (*c*) Y-maze with red lines and shading to show the two target rooms (or boxes) signalled by a cue on the back wall. When swimming forward, the eyes of the animal had to pass the red line to count as an entry. Dimensions of each room 7.5 × 10.3 cm. (*d*) Y-maze set-up for alternating sides training trials (TT; with frozen shrimp next to positive paddle) with a positive and neutral paddle. (*e*) Y-maze set-up for trials for double neutral TT with two neutral paddles. (*f*) Y-maze set-up for trials for ambiguous (AMB) trials with a 45° diagonal line and a neutral paddle. For all trials, ‘correct’ sides were assigned to the left or right box randomly.

Subadult stumpy-spined cuttlefish (*Sepia bandensis*, *n* = 32) were captive-bred and purchased as hatchlings from the Marine Biological Laboratory Center for Cephalopod Culture (Woods Hole, MA, USA). Cuttlefish were fed ad libitum on live mysid shrimp (*Mysidopsis bahia*) until approximately six weeks post-hatching then were fed three live grass shrimp (*Penaeus* spp.) per day. All animals were between two and three months of age at the time of the study and were not sexually dimorphic. Cuttlefish were maintained in a recirculating seawater system (1600 l) held at 23.5–25.5°C, filtered via physical, chemical and biological filtration and reared in floating tub enclosures (30 cm diameter and 8 cm deep) with four to five hatchlings per tub until animals were housed individually for trials. Each housing tub contained a sand and pebble bed and various enrichments (plastic plants, coral rubble, shells and polyvinyl chloride tubes; figure 1*a*,*b*). Experiments were conducted between May 2023 and March 2024.

### (b) Pre-training

For two weeks, cuttlefish were trained to associate a cue (either a horizontal or a vertical striped paddle of equal size and equal pattern dimensions; each animal was trained with only one randomly allocated orientation) with a positive food reward while in home tanks. A paddle placed in the tub with stripes at exactly 180° (horizontal) or 90° (vertical) signalled when food was given. During these two weeks, cuttlefish were also trained to eat thawed, frozen shrimp by first presenting frozen food initially moved about by the experimenter, then simply by dropping thawed shrimp into the tub. All cuttlefish moved onto the next step after showing orientation toward visual cues and hunting behaviour toward frozen shrimp.

Hunting behaviour was defined as orienting the body toward the visual cue, swimming slowly toward it with a postural component of front-raised arms (usually darkened) that were waved side to side [28,29]. We term this suite of behaviours ‘hunting body pattern’ (HBP).

### (c) Training trials

The experimental apparatus was a LANDEN Glass Air Aquarium 1.6-gallon rimless tank. The tank was placed on a stage with two Ulanzi Ultra Bright LED Video Lights providing illumination of the arena from above and each side, with a Sony AX33 4K Handycam recording directly overhead. The tank was turned into a three-chamber Y-maze by adding a removable divider halfway to form a start box and a shorter divider forming two small target boxes (figure 1*c*). At the back of the two-chamber rooms, the positive cue and the neutral cue were placed against the rear wall, with stripes at either 180° or 90° (figure 1*d*).

The cuttlefish was placed in the start room with the middle barrier in place to obstruct the view of the cues. After 2 min of acclimation, the middle barrier was removed, and the cuttlefish could view cues in each room. For the first three trials, whole thawed shrimp were dropped in front of the positive cue to get the attention of the cuttlefish. Positive cue position was randomized to the left or right chamber throughout all trials and tests. For the following trials, two frozen shrimp were partially buried at the back of the reinforced chamber right up against the cue, with the neutral chamber having nothing in the room except the cue paddle. Each trial ran for 10 min or until the cuttlefish successfully ate the shrimp.

Once each subject had completed five training trials (TTs), we began assessing acquisition (the procedure did not change, but we began analysing behaviours for evidence of cue learning), with a criterion that two of the three subsequent trials must be successful (making a total of eight trials minimum all animals completed). Acquisition criteria were (i) cuttlefish must successfully enter the correct (rewarded) side first in two of the last three trials, and (ii) cuttlefish must only search the correct room in two of the last three trials. Cuttlefish that did not reach both criteria in trial 8 were given two additional TTs, and learning was reassessed using the same rules but applied to trials 8, 9 and 10. Twenty-one cuttlefish passed within 10 trials, and 11 were excluded from the experiment and were moved to the retirement or breeding colony.

### (d) Double neutral trials

We used a looser criterion for acquisition and fewer TTs overall than in other operant-conditioning studies using cuttlefish [30,31] because we considered daily handling and testing may have accumulating stress effects. Because ‘stress’ was a treatment in this study that we aimed to control, we used a minimal number of daily TTs but added an additional, single trial as a secondary way of assessing acquisition: a probe trial at the end of training to assess whether learning was specific to the reinforced visual cue. One trial was given with both chambers containing the neutral cue, and no food rewards offered (figure 1*e*). These ‘double neutral’ (DN) trials were otherwise identical to TTs. We considered these trials showed evidence of animals having learned the association if the animals either chose not to enter either room, entered rooms with increased latency compared to correct room entry in the most recent TT or did not show HBP while exploring target rooms. If cuttlefish did not meet two of these three criteria, we gave three further TTs, and the animal must have re-met the training criteria over three successive trials before they were retested on DN trials. Seventeen animals in the study passed the DN phase either on their first or second attempt (12 on first attempt and five on second attempt), and four were excluded at this stage. Before proceeding to the test trials, we gave a single reminder training that was identical to all other TTs.

### (e) Stress experience

After training, animals were divided into treatment and control groups using a random number generator. The treatment group (*n* = 9) was given impoverished housing, removing all enrichments (figure 1*a*, and leaving a bare tub, figure 1*b*) for 6 days before test trials and through the final test trial. Three days before test trials, the treatment group was also chased and repeatedly briefly restrained by an experimenter with a small hand net for 3 min, twice per day. Control animals (*n* = 8) remained undisturbed in their standard enclosures.

### (f) Test trials

Test trials used the same experimental apparatus and timings. The two cues placed in the back of the target rooms were the existing neutral cue and a new ‘ambiguous cue’, with black and white, left-leaning lines at 45° diagonal (i.e. exactly intermediate between the horizontal and vertical cues). Although previous studies have shown evidence for turn bias in choice tasks with cuttlefish [32,33], it has not been shown that animals have an innate preference for visual cue orientation. No frozen shrimp were placed in the end boxes (figure 1*f*).

**Figure 2.**
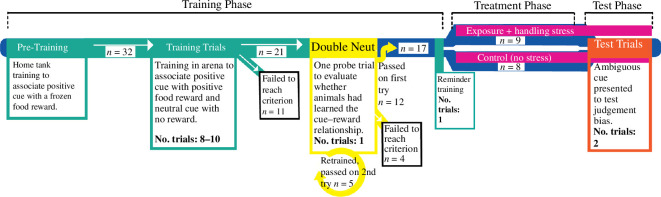
Timeline diagram showing the sequence of trial types and the number of each kind.

Animals were fed directly after every test trial to ensure that any lack of food search behaviour was not due to a lack of food motivation—all animals ate readily once returned to their home tanks. Because performance of learned tasks is inherently noisy in cephalopods [34], we performed two identical test trials over consecutive days for each animal. A timeline diagram of the full experiment is shown in figure 2.

### (g) Data analysis and statistical procedures

From recorded footage, we measured side first entered, latency to enter the target room (the reinforced room during training, either neutral-cue room during the DN trial and the ambiguous-cued room during tests) and duration of time spent exhibiting HBP in each target room (see electronic supplementary material).

‘Side first entered’ was defined as the room the cuttlefish moved into first after the trial was started by the removal of the horizontal barrier. Entry was counted when the animal’s eyes had crossed the ‘room line’ (see figure 1*c*, for room delineation). We used Fisher’s exact tests to compare counts of correct versus incorrect rooms chosen between the two groups. The critical alpha was set at 0.05, and all *p* values were two-tailed.

For latency to choose a correct target box (which we coded as either box in the DN and the ambiguous-cue paired room in the test trials), time started once the middle divider was removed and stopped once cuttlefish eyes crossed the ‘room line’. We analysed data from the last two successful TTs, each animal’s one successful DN trial and for each of the two identical test trials, using a general linear model (GLM) with fixed factors of trial (repeated measures) and treatment (stress versus control) and subject as a random factor. Latencies to target-box entry when the animal stayed in the start room were recorded at 600 s (10 min). To account for this ceiling effect, we applied an arcsine transformation to latency data.

Time spent within a target room while showing HBP was also measured for each test trial. Time was recorded once the animal crossed the ‘room line’ and was expressing HBP. If the animal left the room or the HBP was no longer expressed, the timer was paused and resumed only if both conditions were met again.

Latencies and duration data were analysed using the GLM function in Prism 10.1 (GraphPad, USA). Pre-planned *post-hoc* comparisons among the two fixed factors were compared with *post-hoc t*-tests adjusted for family-wise error rate using the built-in ‘two step’ method of Benjamini, Krieger and Yekutieli. The post-correction critical alpha was set at 0.05, and all *p*-values are two tailed.

## 3. Results

**Figure 3.**
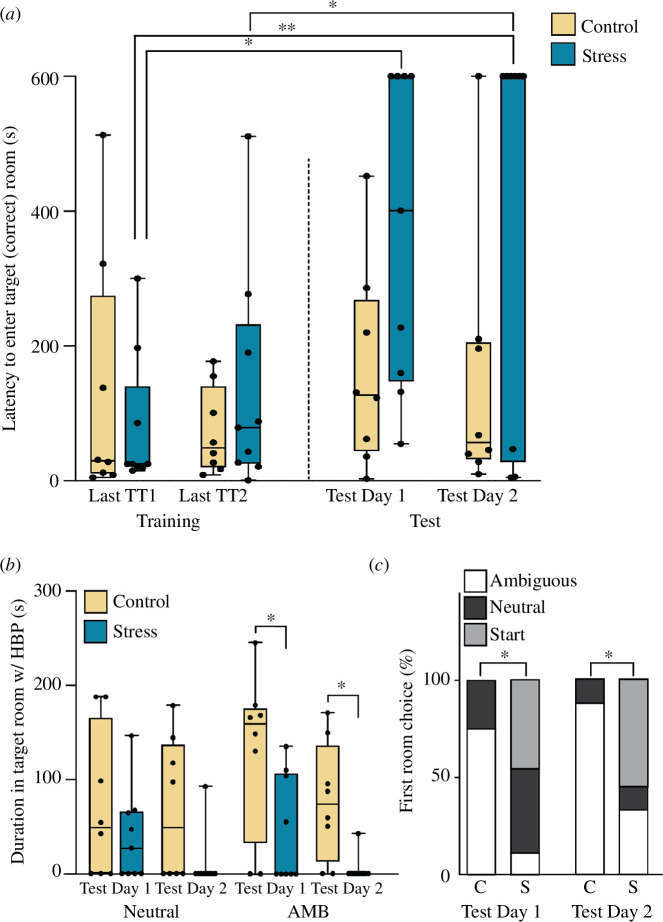
(*a*) Latency to enter a target room in the final two TTs and the two test trials. In test trials conducted after the stress or control treatment, animals in the stressed group showed increased latency to enter either target box (Test Day 1, *p* = 0.01; Test Day 2, *p* = 0.005). (*b*) Total time spent in each room during test trials with HBP. Stress-group cuttlefish spent significantly less time in the ambiguous-cued room than controls on both test days (Test Day 1, *p* = 0.036; Test Day 2, *p* = 0.013), but there were no differences in the durations spent in the neutral room on either day. (*c*) We compared the proportion of animals in each group that chose to enter the room with the ambiguous cue first. On both test days, a significantly greater percentage of control animals (C) chose the ambiguous room (white) to enter first. In contrast, the majority of stressed cuttlefish (S) chose a different room (either never left the start box or entered the neutral room first; Fisher’s exact tests of C versus S, Day 1, *p* = 0.015, Day 2, *p* = 0.049). Boxplots show box boundaries at 25–75 percentiles, whiskers are min–max and lines show the median.

Latency to enter the target room was measured across trials and between groups for the final two TTs and test trials (figure 3*a*). There was an overall significant effect both for trial (*F*_3,44_ = 4.31, *p* = 0.010) and for treatment (*F*_1,15_ = 7.91, *p* = 0.013), as well as a marginal interaction term (Trial × Treatment, *F*_4,60_ = 2.51, *p* = 0.051). Pairwise comparisons across trials within the control group showed significant increases in latency for DN trial versus TT 2 (*p* = 0.046). In the stressed group, there were significant increases in latency to enter between both the test days versus the last TT (Last TT1 versus Test Day 1, *p* = 0.01; Last TT1 versus Test Day 2, *p* = 0.005). Within each trial, latency to enter the target room was lower among control than stressed animals only on the test days (Test Day 1, *p* = 0.032; Test Day 2, *p* = 0.035).

In the test trials, we compared the duration of time spent in each target room when the cuttlefish was expressing HBP. There was an overall effect of treatment (*F*_1,15_ = 6.80, *p* = 0.019). Planned *post-hoc* comparisons showed that stress-group cuttlefish spent significantly less time in the ambiguous-cued room than controls on both test days (Test Day 1, *p* = 0.036; Test Day 2, *p* = 0.013; figure 3*b*), but there were no differences in the durations spent in the neutral room on either day.

For test trials, we also measured the binary outcome of the target room first entered. On Test Day 1, control-group cuttlefish were significantly more likely to enter the ambiguous room first, compared with stressed cuttlefish (figure 3*c*; Fisher’s exact test, *p* = 0.015). The second day of test trials showed similar trends, with control cuttlefish choosing the ambiguous room first significantly more than the treatment group, which chose to either enter the neutral or remain in the start room. We coded these two as ‘incorrect’ choices for the Fisher’s exact test (figure 3*c*; Fisher’s exact test, *p* = 0.049).

## 4. Discussion

Here, we demonstrate that cuttlefish show evidence of negative cognitive bias, the first time this ability has been shown in any cephalopod. The combination of exposure and handling stress likely represents common experiences of laboratory-housed cuttlefish; thus, we suggest that cuttlefish may experience negative affective states as a result of sub-standard and species-inappropriate husbandry.

Our experimental procedure diverges somewhat from typical JBTs. Presentation of the ambiguous cue alongside the negative (neutral) training cue is an unusual ambiguity test used because training also employed a choice test format to facilitate cuttlefish learning. Interpretation is difficult because a well-trained animal perceiving the ambiguous stimulus as intermediate between training cues should anticipate it to be more rewarding than the negative cue and hence prefer it. Preference for the negative cue could thus reflect neophobia and/or poor task learning rather than a negative cognitive bias. However, lower hunting behaviour when in the ambiguous room plus a tendency to not leave the start room at all both point to stressed cuttlefish interpreting ambiguity more negatively than controls. Likewise, while our DN trials were unrewarded, providing food rewards during the DN trial might have more clearly shown cue-specific learning. Despite these differences in procedure, our stress-group animals showed very different responses to the ambiguous cue than control-group animals. Supporting evidence can also be drawn from studies of positive affect in cuttlefish [13] and both positive and negative affect in octopus [12,35], which are closely related to cuttlefish.

Overall, our data indicate that stress has negative effects on cephalopods that are not only physiological [14] but also affective. To our knowledge, this is the first indication of negative judgement bias in cephalopods, adding to the small but growing body of literature suggesting that this capacity for complex processing is not exclusive to vertebrates [10–12,35]. We suggest that JBT-type tasks are useful and novel tools for assessing the welfare of captive cephalopods and for evaluating refinements to their care in laboratory and educational settings.

## Data Availability

Raw data are submitted as an Excel sheet designated as electronic supplementary material [36].
